# The Effect of Exon 7 Deletion during the Evolution of TRIMCyp Fusion Proteins on Viral Restriction, Cytoplasmic Body Formation and Multimerization

**DOI:** 10.1371/journal.pone.0121666

**Published:** 2015-03-30

**Authors:** Feng Liang Liu, Yi Qun Kuang, Dan Mu, Hong Yi Zheng, Jia Wu Zhu, Yong Tang Zheng

**Affiliations:** 1 Key Laboratory of Animal Models and Human Disease Mechanisms of the Chinese Academy of Sciences and Yunnan Province, Kunming Institute of Zoology, Chinese Academy of Sciences, Kunming, Yunnan, China; 2 Kunming College of Life Science, University of the Chinese Academy of Sciences, Kunming, Yunnan, China; 3 University of Science and Technology of China, Hefei, Anhui, China; University of Colorado Denver, UNITED STATES

## Abstract

TRIMCyp is a fusion protein consisting of the *TRIM5* gene product and retrotransposed Cyclophilin A (CypA). Two primate TRIMCyp fusion proteins with varying anti-HIV-1 activities independently evolved in owl monkeys and Old World monkeys. In addition, Old World monkey TRIMCyps lack exon7, which encodes amino acids in the Linker2 region. Previous studies on TRIM5α indicated that this region affects anti-retroviral activity, cytoplasmic body formation, and multimerization. The effects of exon7 deletion on the functions of the TRIMCyp are unclear. In this study, we found that the cytoplasmic bodies and multimers of owl monkey TRIMCyp (omTRIMCyp) are different from those of northern pig-tailed macaque TRIMCyp (npmTRIMCyp). In addition, we demonstrated that exon7 deletion affected cytoplasmic body formation and multimerization. Moreover, we unexpectedly found two chimeric proteins of omTRIMCyp and npmTRIMCyp that failed to block HIV-1 replication, despite the presence of CypA in omTRIMCyp. Further studies indicated that the cytoplasmic bodies and spontaneous multimerization were not responsible for TRIMCyp anti-HIV-1 activity. Moreover, potent viral restriction is associated with higher amounts of monomeric TRIMCyp when the CypA domain is able to recognize and bind to the HIV-1 capsid. Our results suggested that the deletion of exon7 during the evolution of TRIMCyp affected its function.

## Introduction

It is well known that HIV-1 is host restricted and rarely replicates in non-human primates, such as the rhesus macaque (*Macaca mulatta*) and owl monkey (*Aotus trivirgatus*). However, pig-tailed macaques are more susceptible to HIV-1 infection than rhesus macaques [1]. Restriction factors are the predominant reason for the lack of replication in most non-human primates. Indeed, rhesus macaque TRIM5α (rhTRIM5α) and owl monkey TRIMCyp (omTRIMCyp) were first identified as restriction factors that prevent HIV-1 replication in monkey cells [2, 3].

Both TRIM5α and TRIMCyp are members of the tripartite motif (TRIM) family. TRIM family members have an N-terminal RING domain, one or two B-box domains, and a coiled-coil domain [4]. In addition to the tripartite motif, TRIM5α has a C-terminal B30.2 (SPRY) domain, whereas TRIMCyp expresses a C-terminal Cyclophilin A (CypA) domain [2,3]. Sequence analysis of omTRIMCyp has shown that CypA cDNA inserts into the *TRIM5* gene and that alternative splicing leads to TRIMCyp and loss of the B30.2(SPRY) domain [2,5]. Both the B30.2 (SPRY) domain from rhTRIM5α and the CypA domain from omTRIMCyp are able to recognize and bind the HIV-1 capsid [2,5,6]. However, the mechanism of TRIMCyp-mediated HIV-1 restriction may not be identical with that of TRIM5α. For example, both coiled-coil and B30.2 (SPRY) or CypA are essential for potent HIV-1 restriction, but the B-box2 domain is only required for TRIM5α-mediated HIV-1 restriction [7, 8].

**Fig 1:**
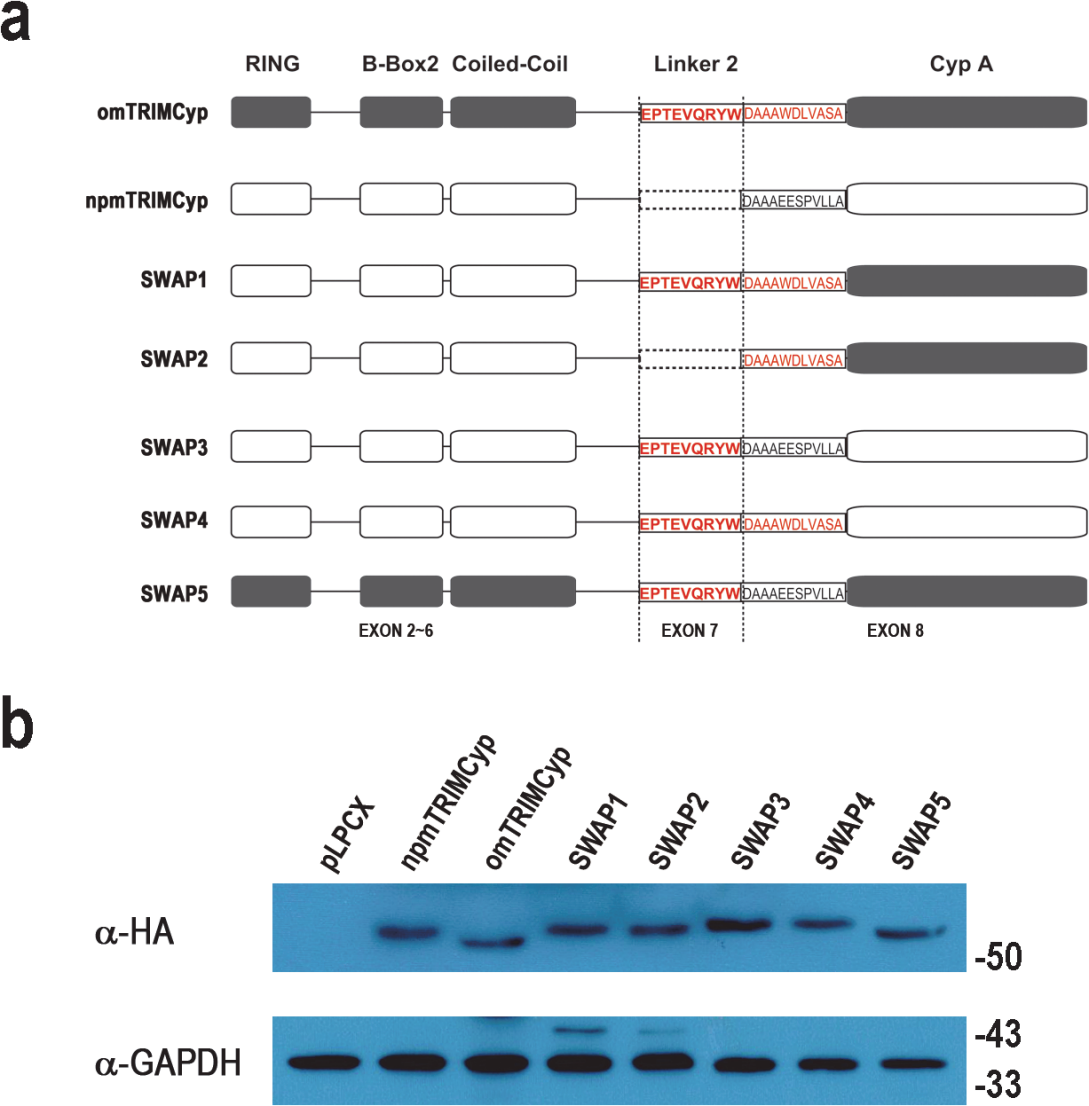
Wild-type and chimeric TRIMCyps. (**a**) A diagram of TRIMCyp proteins is shown. All domains and the Linker2 region are labeled on the top, and all exons encoding TRIMCyp are labeled on the bottom. Dashed lines indicate the boundaries between exon6 and exon7 or exon7 and exon8, respectively. Loss of exon7 is shown by dashed boxes. (**b**) The levels of TRIMCyp protein were analyzed by Western blots. Cell lysates from CRFK cells stably expressing pLPCX or indicated TRIMCyp proteins were analyzed by Western blots immunostained with a mouse anti-HA (top panel) or anti-GAPDH (bottom panel) antibody.

We and others previously found a novel TRIM5-CypA fusion protein widely expressed in Old World monkeys, including northern pig-tailed macaque (*Macaca leonina*), Sunda pig-tailed macaque (*Macaca nemestrina*), rhesus macaque (*Macaca mulatta*), cynomolgus macaque (*Macaca fascicularis*), and Assam macaque (*Macaca assamensis*) [2,5,9–14]. The major difference between Old World monkey TRIMCyps and omTRIMCyp is that the former does not contain the exon7 of *TRIM5* (Fig. 1A), which encodes amino acids in the central domain of the Linker2 region [2, 5, 9–13]. In addition, Old World monkey TRIMCyps cannot restrict HIV-1. It has been demonstrated that a single amino acid mutation in the CypA domain recovered restriction of infection [11, 15, 16]. Therefore, it is a plausible that the CypA domain determines anti-HIV-1 activity.

However, the length of Linker2 significantly affected TRIMCyp-mediated HIV-1 restriction. Neagu *et al* constructed TRIMCyp proteins by fusing human CypA to the Linker2 region or B30.2 (SPRY) domain of human TRIM5α at 11 different positions and found that only three TRIMCyp fusion proteins had anti-HIV-1 activity even though all of them were able to bind the HIV-1 capsid [17]. In addition, Linker2 insertion or deletion also affected the anti-HIV-1 activity of TRIM5α, TRIM family chimeric proteins, and CypA chimeric proteins, but its function remains controversial [18–22]. Old World monkey TRIMCyps lack *TRIM5* exon7, which results in a shorter Linker2 region. The significance of the exon7 deletion during the evolution of Old World monkey TRIMCyps remains to be elucidated. In the present study, we used northern pig-tailed macaque TRIMCyp (npmTRIMCyp) as a model to study the functions of exon7 deletion during the evolution of TRIMCyp. We found that npmTRIMCyp displayed different cytoplasmic body localization and multimerization status compared with omTRIMCyp. Furthermore, our results indicated that exon7 deletion affected cytoplasmic body formation and multimerization. Both cytoplasmic bodies and multimerization did not provide TRIMCyp anti-HIV-1 activity, even though the CypA domain has the ability to bind capsid.

## Materials and Methods

### Cell lines and plasmids

The human embryonic kidney cell line 293T and feline kidney cell line CRFK were obtained from the Shanghai Cell Bank of the Chinese Academy of Sciences and cultured in Dulbecco’s modified Eagle’s medium (DMEM) supplemented with 10% fetal bovine serum.

Recombinant pLPCX expression plasmids of omTRIMCyp and npmTRIMCyp were previously constructed [23]. SWAP1—SWAP5 (Fig. 1A), which are five chimeric proteins of omTRIMCyp and npmTRIMCyp, were constructed by overlapping PCR using a standard protocol. All TRIMCyp proteins encoded by the pLPCX vectors were HA tagged at the C-terminus.

### Creation of cell lines stably expressing TRIMCyp variants

CRFK cell lines stably expressing TRIMCyp variants were created as previously described [9]. In brief, pLPCX plasmids containing TRIMCyp cDNA or control pLPCX plasmid were transfected into CRFK using Lipofectamine 2000 (Invitrogen) in 24-well plates. The media containing the transfection mixtures were removed, and the cells were subcultured at a 1:8 dilution in fresh growth medium twenty-four hours after transfection. Forty-eight hours post-transfection, the selection medium containing 5 μg/ml puromycin (sigma) was added to the medium to select for cell lines stably expressing TRIMCyp variants, and the selection medium was changed every three days until obtaining stable cell lines.

### Western blot

Whole cell lysates were extracted with PBS buffer supplemented with 1% Nonidet P-40, 1 mM EDTA, 1 mM PMSF, and 1× Protease Inhibitor Cocktail (Sigma). After SDS-polyacrylamide gel electrophoresis (SDS-PAGE) in a 5–10% Tris-HCl gel, proteins were transferred onto polyvinylidene fluoride (PVDF) membranes (Millipore). The membranes were blocked with 5% non-fat milk in TBST for 1 hour and incubated overnight with mouse monoclonal anti-HA antibody (1:2,500; cwbiotech) or mouse monoclonal anti-GAPDH antibody (1:15,000; cwbiotech). After washing 3 times with TBST, the membranes were incubated with horseradish peroxidase (HRP)-labeled Goat Anti-Mouse IgG (H+L) antibody (1:20,000; KPL) for 1 hour, and developed using Immobilon Western Chemiluminescent HRP Substrate (Millipore).

### Virus packaging and infectivity assay

VSV-G pseudotyped HIV-1-GFP virus was packaged as previously described [9]. Briefly, pCMV-ΔR8.2 (a plasmid expressing HIV-1 Gag-Pol protein), pCMV-VSV-G (a plasmid expressing VSV-G protein), and pTRIP-GFP (a reporter plasmid expressing GFP protein) were co-transfected into 293T cells using Lipofectamine 2000, and the cells were subsequently incubated overnight. The next day, the medium was changed and the viral supernatants were collected after 48 hours, filtered, and stored at -80°C.

CRFK cells (5×10^4^) seeded in 24-well plates were infected with serial dilutions of VSV-G pseudotyped HIV-1-GFP virus and incubated overnight. Cells were then washed and cultured for an additional 48 hours. GFP-positive cells were counted using a FACSVerse flow cytometer (Becton Dickinson).

### Immunofluorescence and confocal microscopy

CRFK cell lines stably expressing TRIMCyp variants were cultured overnight on cover slips and fixed in ice-cold acetone for 30 minutes. Cells were then washed with PBS buffer, blocked with 2% bovine serum albumin (BSA), and incubated with mouse monoclonal anti-HA antibody (1:1,000; cwbiotech) together with Alexa Fluor 488-labeled Goat Anti-Mouse IgG(H+L) (1:500; Beyotime). Nuclei were stained with DAPI (Sigma) for 5 min. Images were obtained using an Olympus FluoView FV1000 confocal microscope.

### Cross-linking TRIMCyp proteins

CRFK cell lines (3×10^6^) stably expressing TRIMCyp variants were lysed in 300 μl PBS buffer with 1% Nonidet P-40, 1 mM EDTA, 1 mM PMSF and 1× Protease Inhibitor Cocktail (Sigma). After centrifugation at 20,000 × g for 5 minutes at 4°C, 20 μl supernatant containing whole cell protein was incubated with 0, 0.25, 0.5, 1, or 2 mM glutaraldehyde for 3 minutes at 37°C. The reaction was quenched with 2 μl 1 M Tris-HCl (pH 8.0). Samples were subjected to SDS-PAGE using a 5–8% Tris-HCl gel and subsequently analyzed by western blot.

### Data analysis

To quantity the ratio of monomer to multimers of TRIMCyp variants, western blot films were scanned, and the band densities of the monomers, dimers, and trimers were measured using ImageJ software (version 1.47, NIH).

To compare the restriction abilities among different TRIMCyp variants, we defined the percent restriction of TRIMCyp variants as $1-\frac{\%GFP^{+}\;cells\;stably\;expressing\;TRIMCyp}{\%GFP^{+}\;cells\;stably\;expressing\;pLPCX\;empty\;control}$

## Results

### Differences in multimerization state and cytoplasmic body formation between omTRIMCyp and npmTRIMCyp

Unlike omTRIMCyp, npmTRIMCyp lacks exon7, which encodes amino acids in the Linker2 region of TRIMCyp (Fig. 1A). To determine whether this deficit leads to a difference in the multimerization state and subcellular localization, we generated CRFK cell lines that stably express omTRIMCyp or npmTRIMCyp containing a C-terminal HA tag. An HIV-1 infection assay showed that omTRIMCyp strongly inhibited HIV-1 replication, while npmTRIMCyp did not (data not shown). These results were consistent with our previous study using TRIMCyp without protein tag [9].

**Fig 2:**
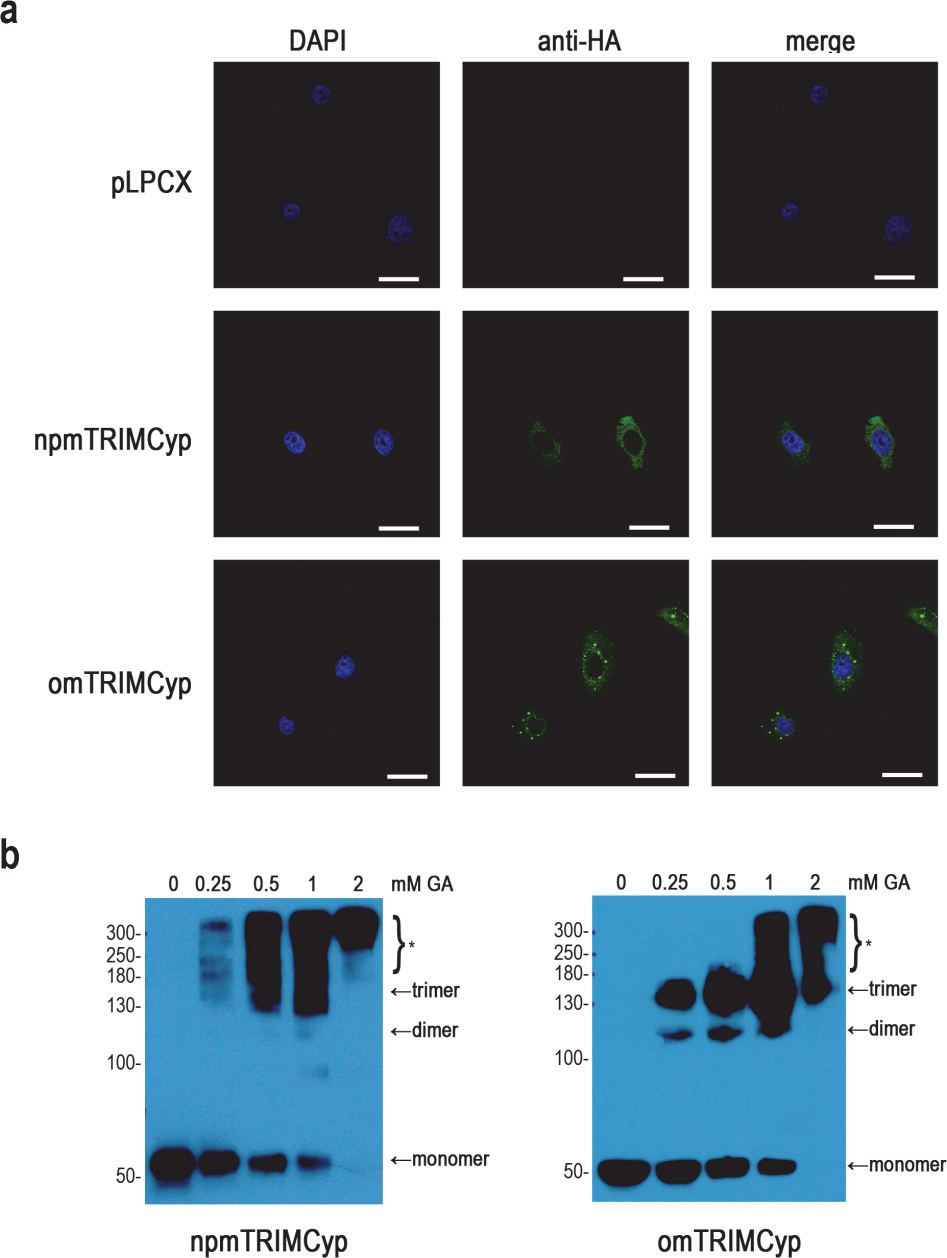
omTRIMCyp and npmTRIMCyp present different status of cytoplasmic bodies and multimerization. (**a**) The subcellular localization of omTRIMCyp and npmTRIMCyp was examined. CRFK cells stably expressing pLPCX or indicated TRIMCyp proteins were fixed and immunostained with a mouse anti-HA antibody (green). All images were obtained using an Olympus FluoView FV1000 confocal microscope. Nuclei were stained with DAPI (blue). The scale bar indicates 50 μm. (**b**) Multimerization of omTRIMCyp and npmTRIMCyp proteins was examined. Lysates from CRFK cells stably expressing TRIMCyp proteins were cross-linked with increasing concentrations of glutaraldehyde (0, 0.25, 0.5, 1, or 2 mM) for 3 minutes at 37°C. The reactions were terminated with the addition of 1 M Tris-HCl (pH 8.0), and cross-linked products were subjected to SDS-PAGE and visualized by Western blot using a mouse anti-HA antibody. Asterisk (*) indicates high-order multimers.

We examined the subcellular localization of omTRIMCyp and npmTRIMCyp. Consistent with previous studies on TRIM5α [20, 24], both omTRIMCyp and npmTRIMCyp were expressed only in the cytoplasm (Fig. 2A). HA-tagged omTRIMCyp formed cytoplasmic bodies, whereas npmTRIMCyp exhibited diffuse cytoplasmic localization (Fig. 2A). We next cross-linked lysates from CRFK cells expressing omTRIMCyp or npmTRIMCyp with increasing concentrations of glutaraldehyde. The omTRIMCyp cross-linked into apparent dimers and trimers at low concentrations of glutaraldehyde (0.25 and 0.5 mM), whereas dimers and trimers of npmTRIMCyp could not be detected easily at all concentrations of glutaraldehyde (Fig. 2B). Thus, omTRIMCyp and npmTRIMCyp exist in different multimerization states and have different localization patterns in the cytoplasmic bodies. Moreover, omTRIMCyp was more prone to form ordered multimers and cytoplasmic bodies than npmTRIMCyp.

### Both multimerization and cytoplasmic bodies are unable to produce TRIMCyp anti-HIV-1 ability

**Fig 3:**
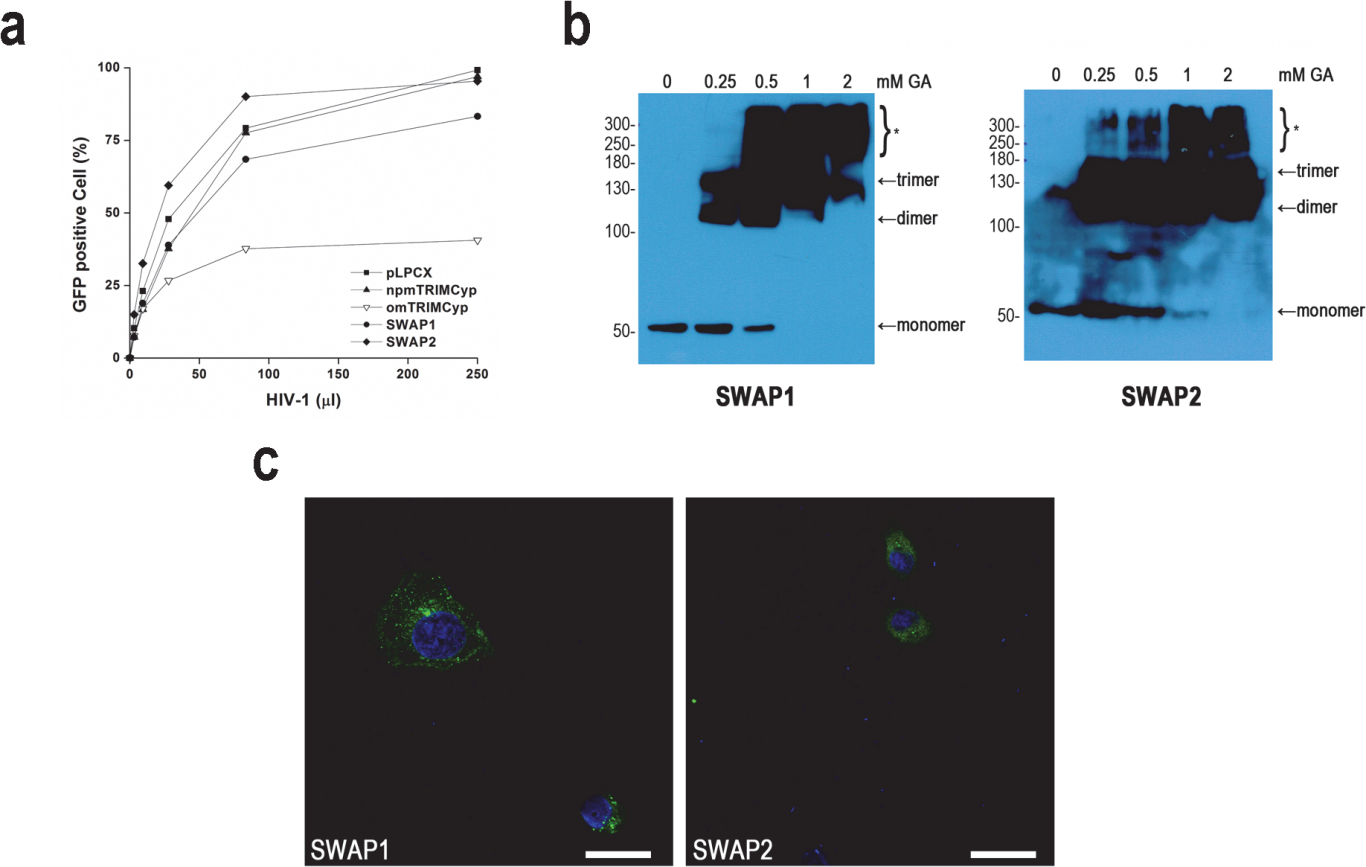
Anti-HIV-1 activity, multimerization, and subcellular localization of SWAP1 and SWAP2. (**a**) Effects of chimeric TRIMCyp proteins on HIV-1 infection. CRFK cells stably expressing pLPCX or indicated TRIMCyp proteins were either mock-infected or infected with serial dilutions of VSV-G pseudotyped HIV-1-GFP virus overnight. Forty-eight hours post-infection, infected cells were sorted by FACS. The results are representative of 4 independent experiments. (**b**) Multimerization of SWAP1 and SWAP2. Lysates from CRFK cells stably expressing TRIMCyp proteins were cross-linked using increasing concentrations of glutaraldehyde (0, 0.25, 0.5, 1, or 2 mM) for 3 minutes at 37°C. The reactions were terminated with the addition of 1 M Tris-HCl (pH 8.0), and cross-linked products were subjected to SDS-PAGE and visualized by Western blot using a mouse anti-HA antibody. Asterisk (*) indicates high-order multimers. Results are representative of 2 independent experiments. (**c**) Subcellular localization of SWAP1 and SWAP2. CRFK cells stably expressing TRIMCyp proteins were fixed and immunostained with a mouse anti-HA antibody (green). All images were obtained using an Olympus FluoView FV1000 confocal microscope. Nuclei were stained with DAPI (blue). The scale bar indicates 50 μm.

Previous studies have indicated that the CypA domain determines TRIMCyp-mediated HIV-1 restriction [11, 15, 16] and that the length of Linker2 also affects anti-HIV-1 activity [17]. To assess the effect of exon7 deletion on TRIMCyp-mediated HIV-1 restriction, we constructed two chimeric proteins, SWAP1 and SWAP2 (Fig. 1A), both of which contain RBCC domains from npmTRIMCyp and the CypA domain from omTRIMCyp, which ensured that artificial TRIMCyp fusion proteins could bind the HIV-1 capsid. SWAP1 includes exon7, while SWAP2 does not (Fig. 1A). Contrary to our expectation, both SWAP1 and SWAP2 lost the HIV-1 restriction potential even though SWAP1 exhibited mild HIV-1 restriction ability (Fig. 3A). Western blot assay indicated that the expression pattern of these recombinant proteins mimicked that of omTRIMCyp (Fig. 1B), which excluded the possibility that low protein expression was the cause of the restriction loss. The result is partly consistent with the finding of Neagu et al that recombinant TRIM and CypA fusion proteins may not inhibit HIV-1 replication [17].

There is evidence indicating that multimerization can provide CypA anti-HIV-1 activity [21,22]. To determine whether the loss of HIV-1 restriction was due to an inability to multimerize, we cross-linked SWAP1 and SWAP2. Both dimers and trimers were easily detected even at low concentrations of glutaraldehyde (0.25 mM) (Fig. 3B). This result suggested that multimerization is not sufficient for HIV-1 restriction.

On the other hand, to test whether cytoplasmic bodies are the cause for the loss of antiviral activity, CRFK cells stably expressing SWAP1 or SWAP2 were immunostained with an anti-HA antibody and analyzed by confocal microscopy. SWAP1 formed apparent cytoplasmic bodies, whereas cytoplasmic bodies were hardly observed with SWAP2 (Fig. 3C). Therefore, these results indicated that the antiviral activity of TRIMCyp was also not dependent on the presence of cytoplasmic bodies.

### Linker2 including exon7 affected the assembly of cytoplasmic bodies and ordered multimers

**Fig 4:**
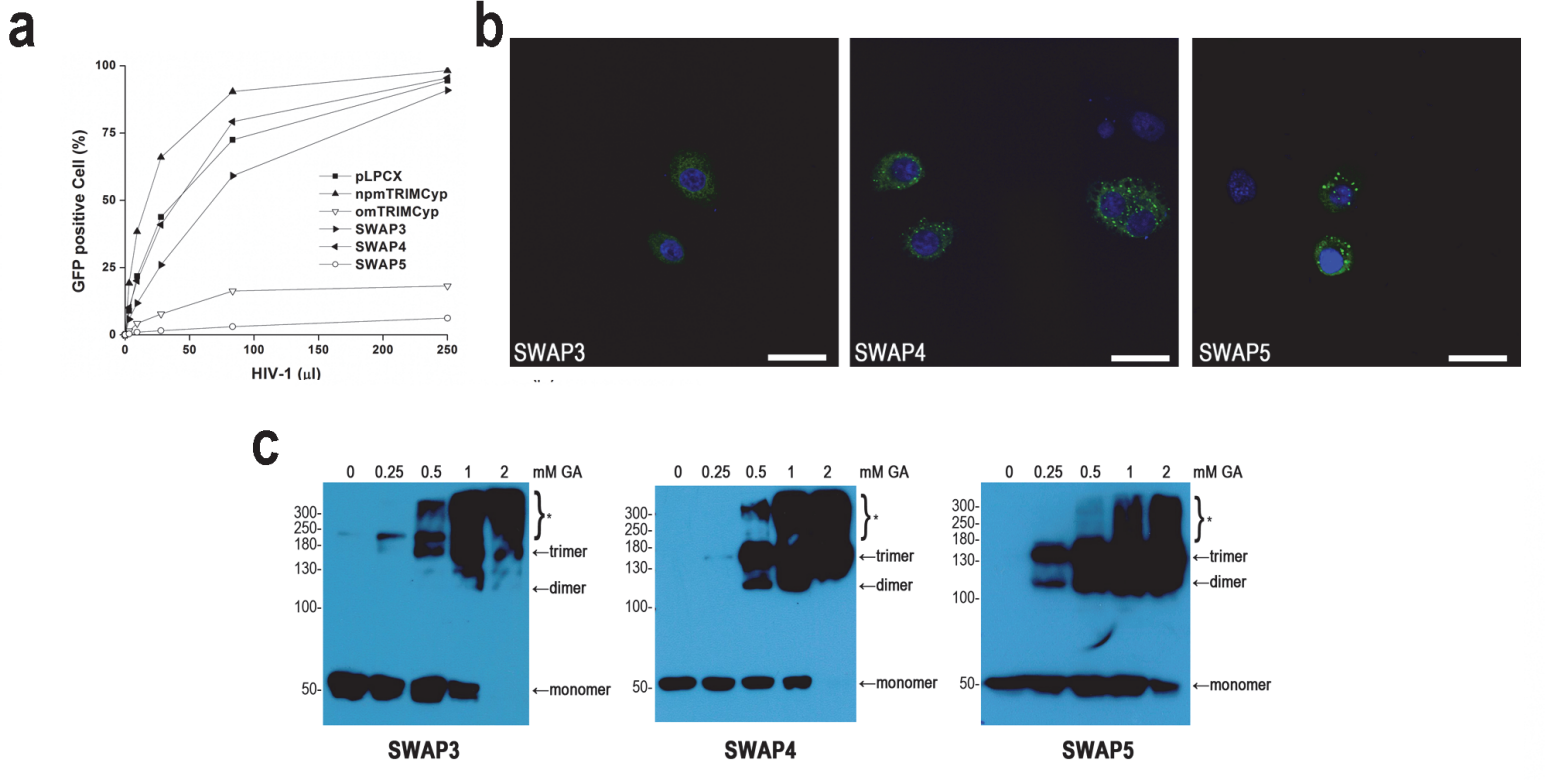
Anti-HIV-1 activity, multimerization, and subcellular localization of SWAP3, SWAP4, and SWAP5. (**a**) Effects of chimeric TRIMCyp proteins on HIV-1 infection. CRFK cells stably expressing pLPCX or TRIMCyp proteins were mock-infected or infected with serial dilutions of VSV-G pseudotyped HIV-1-GFP virus overnight. Forty-eight hours post-infection, infected cells were counted by FACS. The results are representative of 3 independent experiments. (**b**) Subcellular localization of SWAP3, SWAP4, and SWAP5. CRFK cells stably expressing TRIMCyp proteins were fixed and immunostained using a mouse anti-HA antibody (green). All images were obtained using an Olympus FluoView FV1000 confocal microscope. Nuclei were stained with DAPI (blue). The scale bar indicates 50 μm. (**c**) Multimerization of SWAP3, SWAP4, and SWAP5. Lysates from CRFK cells stably expressing the indicated TRIMCyp proteins were cross-linked with increasing concentrations of glutaraldehyde (0, 0.25, 0.5, 1, or 2 mM) for 3 minutes at 37°C. The reaction was terminated with the addition of 1 M Tris-HCl (pH 8.0), and cross-linked products were subjected to SDS-PAGE and visualized by Western blot using a mouse anti-HA antibody. Asterisk (*) indicates high-order multimers. The results are representative of 2 independent experiments.

Both omTRIMCyp and SWAP1, which contained exon7, formed cytoplasmic bodies, whereas npmTRIMCyp and SWAP2, which did not encode exon7, did not (Fig. 2A and 3C). This suggested that exon7 may affect cytoplasmic body formation. In order to evaluate this possibility, we constructed SWAP3, which inserted the omTRIMCyp exon7 sequence into the backbone of npmTRIMCyp (Fig. 1A). As expected, SWAP3 did not inhibit HIV-1 replication (Fig. 4A). However, SWAP3 also failed to form cytoplasmic bodies (Fig. 4B). As shown in Fig. 1A, the main difference between SWAP1 and SWAP3 was the presence of the C-terminus of Linker2 in addition to the CypA domain. To test whether the C-terminus of Linker2 affected cytoplasmic body formation, we also constructed SWAP4 and SWAP5 (Fig. 1A). The subsequent analysis showed that both of them were able to form cytoplasmic bodies very efficiently (Fig. 4B). These results demonstrated that exon7 was required but not sufficient for cytoplasmic body formation and that the C-terminus of Linker2 also affected cytoplasmic body assembly.

The cross-linking assay showed that SWAP3 only formed trimers after incubation with 0.5 mM glutaraldehyde, whereas npmTRIMCyp yielded a smear of proteins at low concentrations of glutaraldehyde (0.25 and 0.5 mM) (Fig. 2B and 4C). Therefore, SWAP3 was able to assemble into more ordered multimers than npmTRIMCyp, which suggested that insertion of exon7 mildly promoted multimerization. Additionally, SWAP4, which was cross-linked into dimers and trimers at low concentrations of glutaraldehyde (0.5 mM), exhibited more ordered multimers than SWAP3 (Fig. 4C), which indicated that the C-terminus of Linker2 also affected multimerization. These results suggested that exon7 was also able to affect the multimerization of npmTRIMCyp. Though we altered the C-terminus of Linker2, SWAP5 did not produce different multimerization status from omTRIMCyp (Fig. 2B and 4C). All results indicated that the Linker2 region including exon7 and the C-terminus of Linker2 was able to affect multimerization.

### The Linker2 region also affected TRIMCyp-mediated HIV-1 restriction

**Fig 5:**
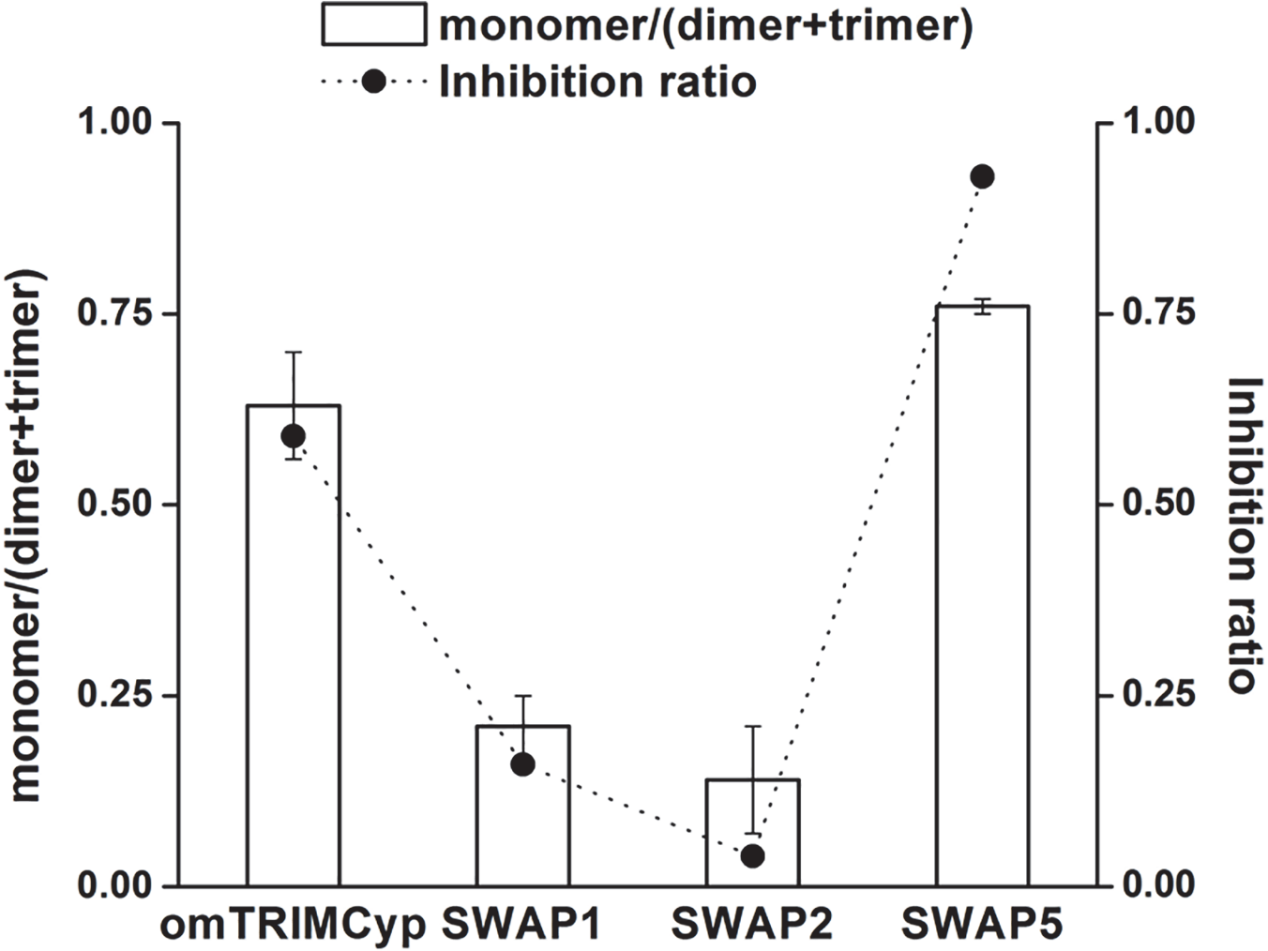
The association between the monomer to multimer ratio and anti-HIV-1 activity. The left y-axis indicates the ratio of monomer to multimers of the indicated TRIMCyp proteins in 0.25 mM glutaraldehyde, which was calculated from the density of monomer, dimer and trimer (Fig. 2B, 3B and 4C) using ImageJ software. The right y-axis indicates the viral restriction abilities of the indicated TRIMCyp proteins (in Fig. 3A and 4A), which was calculated using the formula: $1-\frac{\%GFP^{+}\;cells\;stably\;expressing\;TRIMCyp}{\%GFP^{+}\;cells\;stably\;expressing\;pLPCX\;empty\;control}$. The error bar represents the SEM.

Both omTRIMCyp and SWAP5 had different C-terminus amino acids of Linker2 and were able to assemble into ordered multimers (Fig. 1A, 2B and 4C). In addition, our results indicated that the C-terminus of Linker2 affected multimerization (Fig. 4C). In order to further study the relationship between the C-terminus of Linker2 and multimerization, we calculated the ratio of monomer to multimers after treatment with a low concentration of glutaraldehyde (0.25 mM) and found that SWAP5 exhibited a higher ratio than omTRIMCyp (Fig. 5). This result further demonstrated that the C-terminus of Linker2 also affected multimerization. Meanwhile, the HIV-1 infection assay showed that SWAP5 had stronger anti-HIV-1 activity than omTRIMCyp (Fig. 4A and 5). Therefore, we inferred that TRIMCyp-mediated HIV-1 restriction may be associated with the monomer to multimer ratio. In order to further corroborate this association, we also calculated the ratio from the SWAP1 and SWAP2 data because they also contain the CypA domain from omTRIMCyp, which can bind the HIV-1 capsid. As a result, SWAP2, which exhibited a lower monomer to multimer ratio, also permitted a more robust replication of HIV-1 (Fig. 5). Among 4 TRIMCyps containing CypA from omTRIMCyp, the higher the monomer to multimer ratio was, the more potent the anti-HIV-1 ability (Fig. 5). These results indicated that TRIMCyp-mediated viral restriction was positively associated with a high monomer to multimer ratio. Therefore, these results suggested the possibility that monomeric TRIMCyp may be more important for HIV-1 restriction than spontaneous multimerization.

## Discussion

The npmTRIMCyp protein, unlike omTRIMCyp, lacks exon7 of *TRIM5*, which encodes amino acids in the central domain of the Linker2 region [9]. The Linker2 region has been shown to play a vital role in TRIM5α, TRIM family chimeras, and CypA chimeras [18–21]. However, few studies to date have explored the role of Linker2 in TRIMCyp function, particularly the effect and the significance of exon7 deletion during the evolution of TRIMCyp. In the present study, we found that omTRIMCyp, unlike npmTRIMCyp, formed cytoplasmic bodies and ordered multimers, and the results suggested that the Linker2 region containing exon7 affected cytoplasmic body formation and ordered multimer assembly. In addition, we unexpectedly found (1) that both SWAP1 and SWAP2 failed to restrict HIV-1 replication even though they formed ordered multimer and contained the CypA domain from omTRIMCyp and (2) that a high monomer to multimer ratio was associated with potent anti-HIV-1 activity. Our results also indicated that cytoplasmic bodies and ordered spontaneous multimers are unable to provide TRIMCyp anti-HIV-1 activity.

After finding differences in the multimerization state and cytoplasmic body formation between omTRIMCyp and npmTRIMCyp, we first constructed two recombinant proteins, SWAP1 and SWAP2, to study the effects of exon7 deletion on TRIMCyp function. We hypothesized that exon7 insertion would mildly alter HIV-1 restriction. However, our results showed that both SWAP1 and SWAP2 almost completely lost anti-HIV-1 activities. Our protein expression assay excluded the possibility that low levels of expression of SWAP1 and SWAP2 caused high HIV-1 infection. Consistent with our results, Neagu *et al* found that TRIMCyp failed to restrict HIV-1 replication even though CypA can bind the HIV-1 capsid [17]. In their study, 11 artificial human TRIMCyp fusion proteins were constructed by fusing human CypA to human TRIM5α at 11 different sites located in the Linker2 or B30.2(SPRY) domain, but only three artificial TRIMCyp fusion proteins had potent anti-HIV-1 activity. Consequently, the possibility that human TRIMCyps without anti-HIV-1 activity were unable to bind the HIV-1 capsid was eliminated [17]. However, the reason remained unknown.

Previous evidence indicated that multimerization and cytoplasmic bodies determined TRIM5α- or TRIMCyp-mediated HIV-1 restriction [17,19,21]. However, our results showed that SWAP1 and SWAP2 were able to form multimers and that SWAP1 assembled into cytoplasmic bodies. These results indicated that multimerization and cytoplasmic bodies may not provide TRIMCyp anti-HIV-1 activity. However, compared to SWAP1, SWAP2 that does not contain exon7 could not form cytoplasmic bodies, which suggested that the loss of exon7 may affect cytoplasmic body assembly. Based on this possibility, we inserted omTRIMCyp exon7 into npmTRIMCyp to generate SWAP3. However, SWAP3 also did not form cytoplasmic bodies. The difference between SWAP3 and SWAP1 is the presence of the C-terminus of the Linker2 and CypA domain. In order to assess whether the C-terminus of Linker2 affected cytoplasmic body assembly, we also constructed SWAP4 and SWAP5. As a result, both SWAP4 and SWAP5 assembled into cytoplasmic bodies. Thus, all results from the subcellular localization assay indicated that Linker2 including exon7 was able to change the cytoplasmic body assembly but that exon7 or the C-terminus of Linker2 did not lead to this change alone. Recently, Sastri et al identified in TRIM5α that the N-terminus of Linker2 also affected cytoplasmic body formation, even though their previous study suggested that the central domain of Linker2 was required for cytoplasmic body formation [20,25]. Based on our and Sastri et al’s results, we thought that the Linker2 region may affect cytoplasmic body assembly through changes in the conformation of TRIMCyp or TRIM5α.

The Linker2 region has also been suggested to be necessary for TRIM5α multimerization [19]. We found that npmTRIMCyp, which lacked exon7, did not form apparent multimers. SWAP3 and SWAP4 were slightly able to multimerize, and SWAP4 formed more ordered multimers than SWAP3. These results indicated that Linker2 also affected multimerization. In order to further study the relationship between Linker2 and multimerization, we calculated the ratio of monomer to multimer from omTRIMCyp and SWAP5. SWAP5 exhibited a weaker multimerization ability than omTRIMCyp, which suggested that the C-terminus of Linker2 also affected multimerization. Thus, our results indicated that the Liker2 region including exon7 and the C-terminus of Linker2 might affect TRIMCyp multimerization.

By calculating the monomer to multimer ratio, we unexpectedly found that SWAP5, which had the weaker multimerization ability, can restrict HIV-1 replication more potently. Therefore, we also calculated the monomer to multimer ratios of SWAP1 and SWAP2. As a result, we found a novel association between HIV-1 restriction and multimerization. That is, the higher the monomer to multimer ratio was, the more potent anti-HIV-1 ability. Previous studies indicated that the CypA domain of Old World monkey TRIMCyps was unable to bind the HIV-1 capsid, which was thought to be the reason why Old World monkey TRIMCyps could not restrict HIV-1 replication [11,15]. Consequently, we did not study the correlation of HIV-1 restriction with multimerization among npmTRIMCyp, SWAP3, and SWAP4. Our results indicated that the monomer may be more important for TRIMCyp-mediated HIV-1 restriction. However, this result is not entirely consistent with previous studies [19,21,26–28], which demonstrated that TRIM5α and TRIMCyp were able to form multimers and that multimerization is required for anti-HIV-1 activity. However, previous studies never quantified the relationship between multimerization and HIV-1 restriction. Previous studies only demonstrated that TRIM5α and TRIMCyp could spontaneously assemble into multimers and that the interaction with the HIV-1 capsid promoted the assembly [29]. However, there are still many questions about multimerization that remain to be solved because protein interaction is a dynamic process. For example, we do not know whether spontaneous multimers directly bind the HIV-1 capsid or whether monomers assemble *de novo* into new multimers at the surface of the capsid, which may differ from spontaneous multimerization. If the latter is the case, then monomers are more important for HIV-1 restriction. However, all possibilities need to be further examined.

Our results also suggested that cytoplasmic bodies may be irrelevant for anti-HIV-1 activity, which is consistent with previous studies on TRIM5α [24,30]. However, the function of cytoplasmic bodies in TRIM5α- or TRIMCyp-mediated HIV-1 restriction remains unclear. A previous report suggested that cytoplasmic bodies were highly dynamic structures and that small cytoplasmic bodies aggregate to form larger ones [31]. Perez-Caballero et al found that treatment with sodium butyrate, which increased the expression level of omTRIMCyp, increased cytoplasmic body formation [30]. In our study, we also used an exogenous overexpression system and therefore could not exclude the possibility that TRIMCyp may not form cytoplasmic bodies at physiological levels of expression. We tried to detect TRIMCyp expression in northern pig-tailed macaque PBMCs, but the anti-CypA antibody could hardly detect endogenous levels of TRIMCyp (data not shown), indicating that npmTRIMCyp expression may be low in these cells. TRIM5α has been shown to play a role in innate immune signaling and multimerization is required for RING domain mediated-E3 ubiquitin ligase [32, 33]. Cytoplasmic bodies maybe have one function whereby TRIMCyp may assemble into multimers, such as dimers, and then interact with other signaling proteins in the cytoplasmic bodies. After interacting with other proteins, multimeric TRIMCyp may be capable of E3 ubiquitin ligase activity. Hence, the function of cytoplasmic bodies also needs further investigation.

Two recent structural studies indicated that the coiled-coil domain mediated TRIM5α to form antiparallel dimers, in contrast with a previous hypothesis, and that Linker2 may also contribute to dimerization [34,35]. Hence, in agreement with a similar mechanism of HIV-1 restriction, we hypothesized that (1) Linker2 may also affect the coiled-coil mediated-dimerization or higher-order self-assembly through changes of charges and (2) the length of the Linker2 region also changed the conformation of the CypA domain and affecting its interaction with the HIV-1 capsid. Additional work is needed to test these possibilities.

In conclusion, omTRIMCyp presented differences in cytoplasmic localization and multimerization status compared with npmTRIMCyp, and exon7 affected multimer assembly and cytoplasmic body formation. Additionally, our results suggested that monomeric TRIMCyp may play a more important role in antiviral activity than spontaneous multimerization.
